# Heparin and insulin in the management of hypertriglyceridemia-associated pancreatitis: case series and literature review

**DOI:** 10.1590/2359-3997000000244

**Published:** 2017-01-27

**Authors:** Mohammad Shafi Kuchay, Khalid J. Farooqui, Tarannum Bano, Manoj Khandelwal, Harmandeep Gill, Ambrish Mithal

**Affiliations:** 1 Gurgaon Haryana India Division of Endocrinology and Diabetes, Medanta – The Medicity, Gurgaon, Haryana, India

## Abstract

Severe hypertriglyceridemia accounts for up to 7% of all cases of acute pancreatitis. Heparin and insulin activate lipoprotein lipase (LPL), thereby reducing plasma triglyceride levels. However, the safety and efficacy of heparin and insulin in the treatment of hypertriglyceridemia-associated acute pancreatitis have not been well established yet. We successfully used heparin and insulin as first-line therapy in four consecutive patients with acute pancreatitis secondary to hypertriglyceridemia. In a literature search, we revised almost all reports published to date of patients managed successfully with this combination. Heparin and insulin appear to be a safe, effective, and inexpensive first-line therapy for hypertriglyceridemia-associated acute pancreatitis.

## INTRODUCTION

Hypertriglyceridemia is the third most common cause of acute pancreatitis after alcohol and cholelithiasis (1). The clinical presentation of hypertriglyceridemia-associated pancreatitis (HTGP) is similar to that of acute pancreatitis due to other causes, although the risk of complications with HTGP may be higher (2). There are no standard treatment guidelines for HTGP, and current treatment strategies include standard supportive therapy for the acute pancreatitis (suspension of enteral intake, fluid repletion, and opiate analgesia), rapidly lowering the plasma triglyceride levels and reducing the likelihood of pancreatitis recurrence by eliminating its triggering factors (3). Anecdotal evidence suggests that a combination of heparin and insulin may be an effective first-line therapy for severe HTGP, but the efficacy of this approach has not been well established. We used this combination successfully in four consecutive patients with HTGP and found it to be safe and effective. All published cases of heparin and insulin therapy to control hypertriglyceridemia in HTGP also vouch for the safety and efficacy of this approach.

## CASES

We report in the present study a case series of four patients (three males and one female), aged between 28–46 years, who presented to our hospital between January 2015 and December 2015. All four patients had symptoms attributable to acute pancreatitis with elevated serum triglyceride levels (Table 1). Ultrasonography and contrast-enhanced computed tomography (CECT) of the abdomen confirmed the occurrence of pancreatic inflammatory changes. Serum markers of pancreatitis (serum amylase and lipase) were elevated in all patients. Three patients had diabetes of variable duration (cases 1, 2, and 4) while one patient (case 3) had no diabetes. All patients with diabetes had uncontrolled glycemic levels (mean glycated hemoglobin of 10.3%) and were on oral antidiabetic agents. None of the four patients used alcohol. Patients 1 and 4 had known hypertriglyceridemia for 3 and 4 years, respectively, while patients 2 and 3 were diagnosed with hypertriglyceridemia during the current episode of pancreatitis. Table 2 shows the baseline characteristics of all four patients.

**Table 1:** Laboratory parameters of the patients

	Parameter	Admission	24 hours	48 hours	72 hours	Day 12
Case 1	TG (mg/dL)	5,860	3,416	2,280	1,578	501
	VLDL (mg/dL)	249	260	256	239	100
Case 2	TG (mg/dL)	3,891	1,851	979	686	320
	VLDL (mg/dL)	262	219	157	142	96
Case 3	TG (mg/dL)	1,820	1,011	876	534	221
	VLDL (mg/dL)	202	180	120	84	42
Case 4	TG (mg/dL)	2,430	1,121	992	601	252
	VLDL (mg/dL)	235	192	143	110	82

To convert triglycerides from mg/dL to mmol/L, divide the value by 88.5.

TG: triglycerides; VLDL: very-low-density lipoprotein.

**Table 2:** Baseline characteristics of the cases

Parameters	Case 1	Case 2	Case 3	Case 4
Age (years)/Gender	32/M	38/M	28/F	46/M
Weight (kg)	103	88	63	82
BMI (kg/m^2^)	35.0	31.1	25.0	28.7
Blood glucose at presentation (mg/dL)	540	232	96	298
HbA1c (%) at presentation	11.2	9.2	5.2	10.8
Diabetes duration	3 years	3 years	ND	4 years
History of pancreatitis	Recurrent (third episode)	First episode	First episode	Recurrent (second episode)
Antidiabetic drugs/day	Metformin 2 g/d Glimepiride 4 mg/d	Metformin 1 g/d Gliclazide 80 mg/d	None	Metformin 1 g/d
Antilipidemic drugs	Fenofibrate 145 mg/d	None	None	Atorvastatin 10 mg/d

BMI: body mass index; M: male; F: female; HbA1c: glycated hemoglobin; ND: no diabetes.

The initial management of all patients included suspension of enteral intake and fluid repletion. Regular insulin infusion was started at 2–5 units per hour and gradually increased to 8–12 units per hour depending on the patient’s blood glucose levels. In the patients with diabetes (cases 1, 2, and 4), 5% dextrose with 0.45% normal saline was started when the glycemic levels reached 180 mg/dL in order to maintain these levels within the 140–180 mg/dL range (*i.e.*, glucose clamping until decrease in circulatory triglyceride levels). Patient 3, who had no diabetes, received 5% dextrose simultaneously with insulin infusion to maintain the glycemic levels at 160 ± 20 mg/dL. The mean duration of the insulin infusion was 72 hours (range 48–96 hours). None of the patients developed hypoglycemia.

Activated partial thromboplastin time (aPTT), prothrombin time (PT), international normalized ratio (INR), and complete blood cell count were obtained from all patients at baseline. Heparin was initiated simultaneously with insulin infusion on the first day of admission. Subcutaneous (SC) unfractionated heparin (UFH, 60 U/kg) every 8 hours was initiated in cases 1 and 3, and SC low-molecular-weight heparin (LMWH, enoxaparin, 1 mg/kg) was administered every 12 hours in cases 2 and 4. Heparin was administered for five days in case 1, four days in case 2, and three days in cases 3 and 4. All patients presented full recovery with controlled glycemic and triglyceride levels, as shown in Table 1.

## DISCUSSION

Hypertriglyceridemia may be primary, when it occurs as a familial trait, or secondary to uncontrolled diabetes, obesity, alcohol consumption, or estrogen therapy. Patients with severe hypertriglyceridemia (triglyceride levels > 2,000 mg/dL [> 22.4 mmol/L]) almost always have both the secondary and genetic forms of hypertriglyceridemia. Three of our patients (cases 1, 2, and 4) had type 2 diabetes mellitus (T2DM) with uncontrolled glycemic levels, while case 3 had no known secondary cause for the hypertriglyceridemia.

Severe hypertriglyceridemia (triglyceride levels > 1,000 mg/dL [> 11.2 mmol/L]) requires urgent treatment to reduce the risk of pancreatitis. Identification and elimination of secondary contributing factors are critical in alleviating the ongoing accumulation of triglyceride-rich lipoprotein. For example, control of glycemic levels in patients with uncontrolled diabetes mellitus alleviates the hypertriglyceridemia, as seen in cases 1, 2, and 4. However, a mere control of secondary contributing factor(s) may not be sufficient to control the hypertriglyceridemia in an acute setting such as that in pancreatitis. Anecdotal evidence has shown substantial decreases in serum triglyceride levels following insulin and heparin therapy. LPL is a pivotal enzyme required for the removal of triglycerides from the plasma. Insulin promotes synthesis and activation of LPL, thereby accelerating chylomicron degradation. Heparan sulfate proteoglycan chains normally bind LPL to the capillary endothelium (4). Heparin, administered as a bolus dose, has a stronger affinity for the LPL binding site than heparan sulfate, leading to a dissociation of heparan-LPL complexes from the endothelium to the plasma (5). This surge of “free” LPL is then able to bind to and metabolize lipoproteins at an accelerated rate, thus lowering serum triglyceride levels (6).

Henzen and cols. have found that serum triglyceride levels decreased from a mean of 3,822 mg/dL (43 mmol/L) to 888.8 mg/dL (10 mmol/L) within 2.8 days from the administration of heparin and insulin in five patients with HTGP (7). In our series of four patients, serum TG levels decreased from a mean of 3,500 mg/dL (39.5 mmol/L) to 849.7 mg/dL (9.6 mmol/L) within 72 hours, which is comparable with the report by Henzen and cols. All patients had a rapid clinical resolution of the pancreatitis. Similarly, Berger and cols. reported a successful HTGP treatment with insulin and heparin in five patients (8). Serum triglyceride levels in their series decreased to < 500 mg/dL (5.6 mmol/L) within 3 days in all cases. Other authors have also reported successful treatment with heparin and insulin in patients with HTGP (Table 3) (9-15). No complications from this treatment regimen were observed in our patients or reported in previously published cases.

**Table 3:** Summary of case reports of hypertriglyceridemia-associated acute pancreatitis managed with insulin and heparin

Case report	Year of publication	Number of patients	Initial triglyceride level (mg/dL)	Treatment used
Henzen and cols.^7^	1999	5	1,323-7,236	Insulin, heparin
Berger and cols.^8^	2001	5	1,590-8,690	Insulin, heparin
Monga and cols.^9^	2003	1	7900	Insulin, heparin
Alagozlu and cols.^10^	2006	1	1,707	Insulin, heparin
Jain and cols.^11^	2007	2	1,808-3,743	Insulin, heparin, fenofibrate
Jain and cols.^12^	2009	1	10,320	Insulin, heparin
Twilla and Mancell^13^	2012	1	5,366	Insulin, heparin, gemfibrozil
Patel^14^	2012	1	> 5,000	Insulin, heparin, fenofibrate, fish oil
Kota and cols.^15^	2014	1	2,080	insulin, heparin
Current study	2015	4	1,820-5,860	Insulin, heparin fenofibrate

For the reports with more than one cases, the value shown for triglycerides correspond to the range of the reported values.

Treatment of severe hypertriglyceridemia with heparin is controversial due to a transient rise in LPL followed by increased degradation and depletion of plasma stores resulting in LPL deficiency (16,17). However, in all case reports, including ours, the triglyceride levels have been maintained low, without recurrence of the hypertriglyceridemia. Whether this concept of depletion of LPL levels after heparin therapy is clinically relevant in HTGP is to be determined.

Heparin and insulin have both been used also as monotherapy in the treatment of severe hypertriglyceridemia in previous studies (18-21). However, more dramatic results have been achieved when both were used in combination (7-15). Plasma exchange (plasmapheresis) has also been used successfully to treat severe HTGP (22); however, this treatment is far more expensive and may not be available at all health care centers. Plasma exchange may be useful in pregnant patients who fail to respond to other forms of treatment (22).

At present, no studies have been conducted to identify the best route (intravenous or SC) to administer heparin and insulin to treat HTGP. Both Henzen and cols. and Berger and cols., in their series of five patients each, used heparin and insulin intravenously in a continuous infusion. The heparin dose was guided by usual parameters of blood coagulation, and the insulin dose by serial blood glucose monitoring (7,8). Jain and cols. published a case report in which SC heparin was used in one patient (11). We also used SC UFH every 8 hours in two of our patients (cases 1 and 3) and SC LMWH every 12 hours in cases 2 and 4. Both SC and intravenous heparin have been used successfully to treat HTGP. On the other hand, insulin has been used uniformly as a continuous infusion in all case series and reports.

The dose of heparin for HTGP management has also not been determined by the studies. Based on anecdotal evidence, we initiated UFH at a dosage based on the patients’ weights (60 U/kg) in cases 1 and 3. In cases 2 and 4, LMWH was administered at a dose of 1 mg/kg. Whether this is the optimal dose to lower serum triglyceride levels remains to be determined in future studies. There is also no consensus in regard to the type of heparin to be used. Both UFH and LMWH have been used and seem to have similar efficacy. None of the studies have reported complications with heparin therapy. In our series, none of the patients had coagulation defects or platelet abnormalities at baseline. Furthermore, none of our patients developed bleeding or platelet complications during treatment. We maintained the INR in all patients ≤ 3.

In conclusion, heparin and insulin appear to be an effective therapy in the management of HTGP. Bleeding is a risk to be considered in this treatment, although no problems were observed in this regard. There is a definite need for clinical guidelines for HTGP management, as none exist to date.
